# Creating Digital Watermarks in Bitmap Images Using Lagrange Interpolation and Bezier Curves

**DOI:** 10.3390/jimaging9100206

**Published:** 2023-09-29

**Authors:** Aigerim Yerimbetova, Elmira Daiyrbayeva, Ekaterina Merzlyakova, Andrey Fionov, Nazerke Baisholan, Mussa Turdalyuly, Nurzhan Mukazhanov, Almas Turganbayev

**Affiliations:** 1 Institute of Information and Computational Technologies, Committee of Science of the Ministry of Education and Science of the Republic of Kazakhstan, Almaty 050010, Kazakhstan; aigerian@mail.ru (A.Y.); m1challenge@inbox.ru (A.T.); 2 Institute of Automation and Information Technologies, Satbayev University, Almaty 050013, Kazakhstan; m.turdalyuly@gmail.com (M.T.); n.mukazhanov@satbayev.university (N.M.); 3 Institute of Automation and Telecommunications, Academy of Logistics and Transport, Almaty 050012, Kazakhstan; 4 Department of Applied Mathematics and Cybernetics, Siberian State University of Telecommunications and Information Sciences, Novosibirsk 630102, Russia; katerina.artist@yandex.ru (E.M.); fionov@sibguti.ru (A.F.); 5 Department of Information Systems, Al-Farabi Kazakh National University, Almaty 050023, Kazakhstan; baisholan@gmail.com

**Keywords:** steganography, digital watermark, Bezier curve, secret message, interpolation, steganalysis

## Abstract

The article is devoted to the introduction of digital watermarks, which formthe basis for copyright protection systems. Methods in this area are aimed at embedding hidden markers that are resistant to various container transformations. This paper proposes a method for embedding a digital watermark into bitmap images using Lagrange interpolation and the Bezier curve formula for five points, called Lagrange interpolation along the Bezier curve 5 (LIBC5). As a means of steganalysis, the RS method was used, which uses a sensitive method of double statistics obtained on the basis of spatial correlations in images. The output value of the RS analysis is the estimated length of the message in the image under study. The stability of the developed LIBC5 method to the detection of message transmission by the RS method has been experimentally determined. The developed method proved to be resistant to RS analysis. A study of the LIBC5 method showed an improvement in quilting resistance compared to that of the INMI image embedding method, which also uses Lagrange interpolation. Thus, the LIBC5 stegosystem can be successfully used to protect confidential data and copyrights.

## 1. Introduction

The construction of digital watermark systems is one of the directions of steganography, the purpose of which is to conceal data in digital media or files so that the embedded mark would be difficult to remove or detect. Unlike the usual task of steganography, i.e., the problem of hiding the fact of data transmission, it is usually not necessary to hide the presence of a watermark in most applications. It is believed that it is possible to predict in advance whether a watermark is contained or not. The scope of applications for digital watermarks includes copyright protection and protection against illegal copying. Various features of the classification of digital watermarks (DWMs) are considered in [1,2,3]: DWM sare classified according to reliability, visibility, capacity and embedding methods. In this paper, we focus on inconspicuous watermarks.

A digital watermark is called invisible if the source signal and the output signal are visually indistinguishable. In this case, an interpolated bitmap image acts as the source signal, and an image with embedded information acts as the output signal.

During the study, the following works were analyzed concerning the issues of interpolation, digital watermarks and application of the Bezier curve:

Onearticle presents studies of the current state and development of digital steganography. A review of the subject area is conducted, and the basic concepts of steganography are described. The principles of digital methods and the purpose of digital watermarks are studied. The article discusses the use of digital watermarks. The difference between the task of hidden data transmission and the task of embedding digital watermarks is shown [4].

Abook presents an exposition of the basic concepts of steganography, as well as a brief description of known stegosystems. The authors describe the main criteria for the stability of stegosystems, including a new criterion proposed by the authors. New results of the work of the research group on the detection of stegosystems for still images, embedding in digital documents and stegosystems for channels with noise, as well as methods for minimizing the detectability of stegosystems in blind stegoanalysis, are presented [5].

In onepaper, the authors propose a new approach to shape interpolation based on the Poisson equation. The authors propose a method of interpolation of a nonlinear gradient field that takes into account both the coordinates of the vertices and the orientation of the surface. When proper boundary conditions are met, intermediate shapes are restored implicitly from interpolated gradient fields, while traditional methods usually manipulate vertex coordinates directly. Experimental results demonstrate that this method avoids the shrinkage problem that occurs during linear shape interpolation [6].

In anotherwork, the methods of interpolation of regions proposed in the literature on databases of moving objects impose restrictions that can have a significant impact on the representation of the evolution of moving regions, in particular when there is a turn between two observations. In this paper, the authors propose a data model for moving regions that allows moving segments to rotate and change their length during their evolution between two observations and uses square Bezier curves to determine the trajectories of their endpoints. Experimental results show that the strategy can be used in addition to the methods of interpolation of regions [7].

In anotherarticle, the researchers present an interpreted fuzzy logic model for edge detection based on a new generalized domain-independent parametric adaptive membership function based on the Bezier curve (ABCMF) constructed for image blurring. In order to create a reliable fuzzy structure using the developed new membership function, the blurred image is convoluted using a simplified edge detector based on fuzziness to determine the direction of intensity changes. From the estimated indicators, the authors conclude that the proposed method provides stable accuracy exceeding 91% compared to its analogues [8].

### Related Work

The following paper discusses the use of the Bezier curve to ensure driving safety, comfort, stability and high mobility of an emergency rescue vehicle, as well as taking into account the actual characteristics of emergency rescue vehicles, such as a large width of the vehicle, a high center of gravity and a large turning radius. The authors have developed a method of trajectory and velocity planning for collision avoidance based on a segmented three-dimensional quadratic Bezier curve. The results presented in the article show that the proposed method of trajectory and speed planning based on a segmented three-dimensional quadratic Bezier curve can effectively improve the mobility of trajectory planning results, increase the efficiency of lane change and reduce lane change time while ensuring the design goals of safety, comfort and stability of the vehicle [9].

Also, methods of hiding information in the spatial domain of imagesinclude methods based on matrix transformations of pixel blocks [10,11].

In [12], it was proposed for the first time to use interpolation methods for image steganography in order to improve the quality of the container. Following this idea, the image is first interpolated, and then the resulting container is used to embed the data. Later, this method was used in the development of steganographic methods such as NMI [12], INP [13], NIE [14], the reversible method of hiding information [15], the method of reversible hiding of data in medical images [16], the method of reversible hiding for interpolation images using the strategy of multilayer bending in the center [17] and others. In some works, the embedding of the date into the container is considered as the embedding of noise. From this point of view, the approach using discrete chaotic maps [18] as a class of masking circuitsis interesting. Such means can be applied for maskingan image and also for encryption systems.

## 2. Background on Lagrange Interpolation and Bezier Curves

In our work, we consider bitmap stegocontainers represented by bmp files. The proposed algorithm is also applicable to other formats that use non-distorting compression methods: tiff, png, pcx, tga, pgm. These containers are matrices of pixel brightness into which copyright information can be recorded.Unlike the known methods of embedding information in images, we propose to initially transform an empty stegocontainer using Lagrange interpolation. Thus, the bitmap can be enlarged by two times so that in addition to the original pixels, there are interpolated values. Let’s take a closer look at how Lagrange interpolation works in a container transform.

Lagrange interpolation is a common method of signal and image processing [19,20,21,22]. In the segment a≤x≤b at certain points $x_{k}$, (node points) k=0,1,…,n, the values of the function f(x) are known. The form of the polynomial of order n, $L_{n}(x)$, whose values at the node points are equal to the values of the function f(x), is:

(1) $$L_{n}(x_{i})=f(x_{i}),i=0,1,\ldots ,n$$

And polynomial $L_{n}(x)$:

(2) $$L_{n}(x)=a_{0}+a_{1}x+\ldots +a_{n}x^{n}$$

is called an interpolation polynomial which is constructed from points $x_{i},i=0,1,\ldots ,n$. Using the Lagrange interpolation formula, it is possible to write $L_{n}(x)$ as a linear combination of the interpolation values of the f(x) at node points:

(3) $$L_{n}(x)=\sum_{k=0}^{n}C_{k}(x)f(x_{k})$$

Considering all the relevant conditions [23], it can be seen that $C_{k}(x)$ is determined by the formula:

(4) $$C_{k}(x)=\frac{\underset{j\neq k}{\prod }(x-x_{j})}{\underset{j\neq k}{\prod }(x_{k}-x_{j})}$$

Thus, the Lagrange interpolation polynomial has the following form:

(5) $$L_{n}(x)=\sum_{k=0}^{n}\frac{\underset{j\neq k}{\prod }(x-x_{j})}{\underset{j\neq k}{\prod }(x_{k}-x_{j})}f(x_{k})$$

To solve the problem of watermark creation, we divide the pixels of the interpolated image into groups of five points, on which the Bezier curve is built using this formula:

(6) $$P(t)=\sum_{i=0}^{n}P_{i}\cdot \frac{n!}{i!(n-1)!}\cdot {\left(1-t\right)}^{n-i}\cdot t^{i}$$

in which *n* = 4, *P(t)* is the value of the Bezier curve point, $P_{i}$ is image pixel value and t∈[0,1]. The step for t can take values of 0.1 or less. The smaller the step value, the more curve values we obtain.

The curves in question were first presented to the general audience in 1962 by Pierre Bezier, who used them for the computer-aided design of automobile bodies. The curves were named after Bezier, and the recursive method of defining curves was named after de Casteljou (de Casteljau’s algorithm). To date, Bezier curve formulas are used in many branches of science, and many scientists are also conducting research in this area [24,25,26,27].

The Bezier curve is a special case of a Bernstein polynomial, which is a parametric curve and defined by the following expression:

(7) $$B(t)=\sum_{i=0}^{n}P_{i}b_{i,n}(t),0\leq t\leq 1,$$

in which n is a number of reference points, i is the reference point number, t is the step, P is the coordinate of the reference point and b(t) is the basis function of the Bezier curve. Then the coordinates of the curve are described depending on the parameter t∈[0,1] in the following way:

For two points: $P=(1-t)P_{1}+tP_{2}$.
For three points: $P={\left(1-t\right)}^{2}P_{1}+2(1-t)tP_{2}+t^{2}P_{3}$.
For four points: $P={\left(1-t\right)}^{3}P_{1}+3{\left(1-t\right)}^{2}tP_{2}+3(1-t)t^{2}P_{3}+t^{3}P_{4}$.
For five points: $P={\left(1-t\right)}^{4}\cdot P_{1}+4\cdot {\left(1-t\right)}^{3}\cdot t\cdot P_{2}+6\cdot {\left(1-t\right)}^{2}\cdot t^{2}\cdot P_{3}+4\cdot (1-t)\cdot t^{3}\cdot P_{4}+t^{4}\cdot P_{5}$

## 3. Embedding Method LIBC5

We call the proposed algorithm LIBC5 (Lagrange interpolation Bezier curve for 5 points). The algorithm is arranged as follows. First, consider a group of five pixel brightness values of an interpolated bitmap image $\left(P_{0},P_{1},P_{2},P_{3},P_{4}\right)$. The values $P_{0},P_{2},P_{4}$ are the pixel values of the original image for the single selected color component (R, G or B), and the values $P_{1}$ and $P_{3}$ are added by interpolation. Embedding a bit of information occurs in pixels $P_{1}$ and $P_{3}$ by taking the nearest point with a Bezier curve rounded to an integer, such that the lowest bit of its value coincides with the bit of information we want to embed. To select the desired value of the Bezier curve point, it is necessary to set such a step t thatprovides a sufficient choice of the values of the curve points.

**Figure 1:**
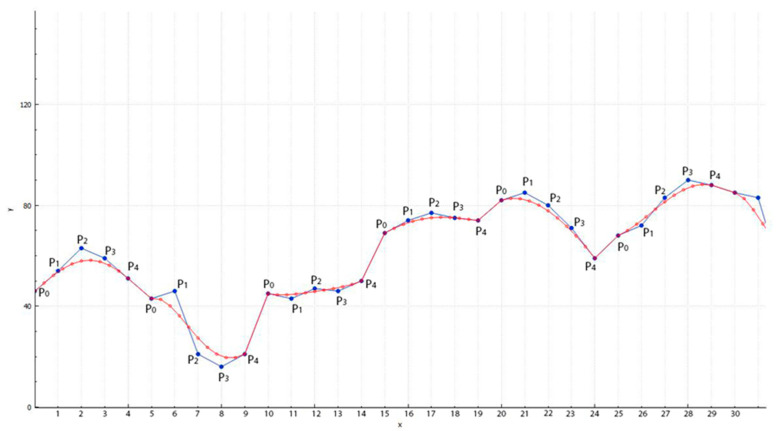
Construction of the Bezier curve for the 029.bmp file with step t=0.1.

Nowconsider the graph of the averaged pixel brightness and the constructed Bezier curve with step t=0.1 (Figure 1):

Along the Y-axis are the brightness values of the image of one of the color component pixels taken from the first row of the pixel matrix in order. The X-axis shows the linear order of pixels and their ordinal numbers. For clarity, in this example, the step was taken as *t* = 0.1. The graph shows that for every 5 brightness points of the interpolated image (blue line), we have 11 points of the Bezier curve (red line).

**Figure 2:**
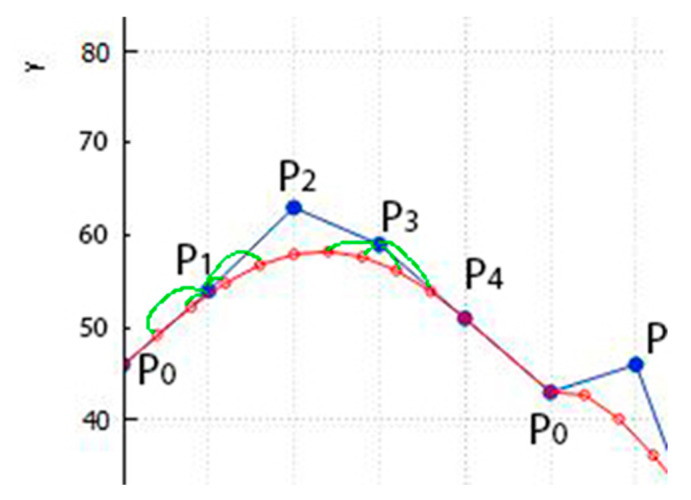
Bezier curve points (on red line) to replace pixels (on blue line) $P_{1}$ and $P_{3}$ for a image file with a step of t=0.1. The y-axis indicates the brightness of the image points.

Bits of information are written to $P_{1}$ and to $P_{3}$ by taking such a value from the Bezier curve, in which the lowest bit is equal to the embedded bit of the message. Figure 2 shows how the corresponding values for replacing pixels $P_{1}$ and $P_{3}$ are determined.

The graph in Figure 2 shows that for point $P_{1}$, there is a choice of four values of the points the Bezier curve, which can be used by rounding them to an integer value and using their lowest bit. It is necessary to take from the Bezier curve the closest value with the point $P_{1}$ such that the lowest bit of this value will coincide with the bit of the embedded message. Then, for point $P_{2}$, we also need to choose a suitable value with a Bezier curve from four possible ones, after which a segment of the Bezier curve is constructed at the next five pixel points and the actions are repeated.

So, let’s denote the rounded values of the Bezier curve which can be used to replace the current point $P_{1}$ or $P_{3}$ by the sets $R_{1}=\{r_{1},\ldots ,r_{k}\}$ and $R_{3}=\{r_{1},\ldots ,r_{k}\}$, respectively, where k is the number of values at a given step t. The value of k actually represents the number of segments into which the curve passing from point $P_{0}$ to point $P_{4}$ is divided, with the exception of the first and last segments, and with the exception of those two segments that surround the value of the curve corresponding to $P_{2}$ in order. Thus, to calculate k, we need to divide the unit segment by the length of the interval t, subtract four segments and divide the resulting value in half to replace the two values $P_{1}$ and $P_{3}$. Then, we need to add one, since in the end we do not need to get the number of segments, but the number of points obtained:

(8) $$k=\frac{1/t-4}{2}+1$$

Simplifying this expression, we obtain a formula for calculating k:

(9) $$k=\frac{1}{2\cdot t}-1$$

Thus, we see that at t=0.1, a choice of four possible values is created. The edge values on the curve corresponding to $P_{0}$, $P_{2}$ and $P_{4}$ are not used to replace the values of $P_{1}$ and $P_{3}$ because they correspond to those pixels of the image that originally formed the pixel matrix before the interpolation process.

**Figure 3:**
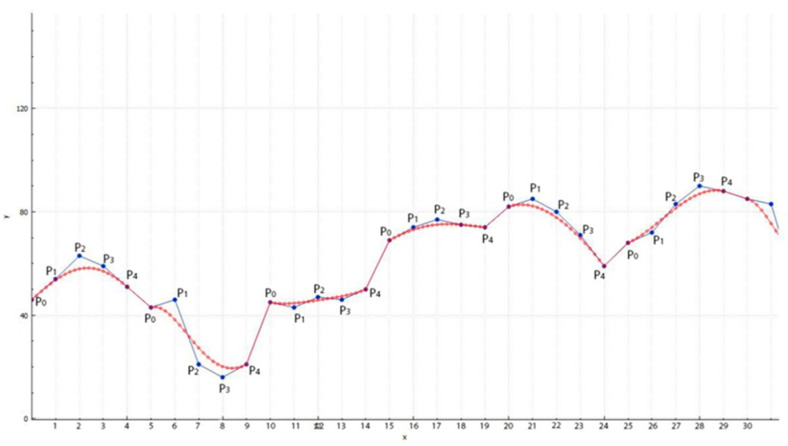
Construction of the Bezier curve for the 029.bmp file with step of *t* = 0.05.

**Figure 4:**
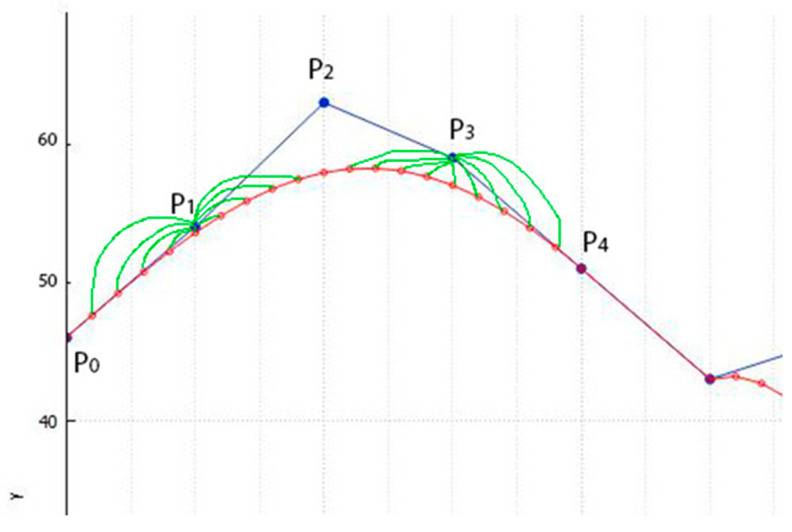
Possible points (on blue line) to replace $P_{1}$ and $P_{3}$ on the Bezier curve (red line) for the 029.bmp file with a step of t=0.05.

Since the values of the points of the Bezier curve must be rounded to an integer, we can get the same values, which will reduce the choice for replacing $P_{1}$ and $P_{3}$. Therefore, we next consider the case at t=0.05. In Figure 3, the red line shows the Bezier curve, and the blue line plots the brightness of the current bitmap image:

Inconstructing a Bezier curve with a step of *t* = 0.05, we have a choice of k = 9 values to replace each of $P_{1}$ and $P_{3}$. Figure 4 shows the possible points to replace $P_{1}$ and $P_{3}$.

Let’s consider an example of embedding a message with the author’s information in a 029.bmp image file using the LIBC5 method considered. Table 1 shows the rounded values for the corresponding *t* values for the 029.bmp file, as well as the correspondence of these values to points $P_{0},P_{1},P_{2},P_{3}$ and $P_{4}$:

Table 1 shows that the following options are available to replace the value of $P_{1}$: 48, 49, 51, 52, 54, 55, 56 and 57, along withoptions such as 58, 57, 56, 55, 54 and 53 to replace $P_{3}$. It should also be taken into account that of the possible values to replace $P_{1}$ and $P_{3}$, we have to choose one suitable value, the lowest bit of which will coincide with the message bit for embedding. At the same time, if the lowest bit of $P_{1}$ and $P_{3}$ has already coincided with the message bit, then no replacement is required.

If we assume that the sequence of message bits is m = (1, 0), we canconvert to binary the values of the set $R_{1}$, corresponding to $P_{1}$:

$r_{1}$=48_10_=110000_2_
$r_{3}$=49_10_=110001_2_
$r_{4}$=51_10_=110011_2_
$r_{5}$=52_10_=110100_2_
$r_{6}$=54_10_=110110_2_
$r_{7}$=55_10_=110111_2_
$r_{8}$=56_10_=111000_2_
$r_{9}$=57_10_=111001_2_

To embed the first bit from the sequence m, we need values from $R_{1}$ whose lowest bit is 1, that is, $r_{3},\;r_{4},\;r_{7}$ and $r_{9}$. Replacing the pixel values for $P_{1}$ with any of them, we write bit 1 into the pixel matrix of the image file and proceed to $P_{3}$. 

For $P_{3}$, we convert the values of the corresponding set $R_{3}$ to binary, excluding duplicate values:

$r_{1}$=58_10_=111010_2_
$r_{3}$=57_10_=111001_2_
$r_{4}$=56_10_=111000_2_
$r_{5}$=55_10_=110111_2_
$r_{6}$=54_10_=110110_2_
$r_{7}$=53_10_=110101_2_

To embed the second bit from sequence m, we need those values from $R_{3}$ that have the least significant bit equal to 0, that is, $r_{1},\;r_{4}$ and $r_{6}$. Replacing the values of the $P_{3}$ pixel with any of these, we write bit 0 inthe pixel matrix of the image file. Hence, a block of five pixels has been passed, and two bits of the message m are written to it.

Similarly, embedding into subsequent blocks of the image, taken sequentially from the pixel matrix, can occur. The 029.bmp image with dimensions of 450 in width and 450 in height allows us to use 90 blocks in each row for each of the RGB components. Thus, the total number of blocks for this image will be 40,500 when using one of the component pixels, or 121,500 when using three components. When embedding two bits in each block, we get the formula (Equation (10)) for calculating the maximum number of embedded bits in a bitmap image by one of the components of the dimensions W in width and H in height by the proposed embedding method:

(10) N=2⋅[W/5]⋅H

In the example considered, *N* = 81,000 bits or 10,125 bytes. If all the components of the pixel brightness are used, the value of N is multiplied by three. It should be noted that if it is necessary to fill the container with half of its capacity, you can use blocks of pixels through one, since the Bezier curve for five points is built separately for each block of five pixels.

If we return to the example considered, the question naturally arises: by what algorithm is the $r_{i}$ value selected to replace an image pixel if we have a sample of several suitable values? This algorithm was implemented in such a way that the first in order is selected from a suitable sample of $r_{i}$ values and used to replace the pixel. So, when replacing $P_{1}$, the value of the Bezier curve point closest to $P_{0}$ from the set of suitable values is used.

Next, we candenote the set of suitable values $r_{i}$ for embedding each next bit $m_{j}$ as $S_{1}=\{s_{1},\ldots ,s_{k-e}\}$ and $S_{3}=\{s_{1},\ldots ,s_{k-f}\}$; that is, $S_{1}$ contains values from $R_{1}$ and $S_{3}$ contains values from $R_{3}$ such that their lowest bit is the same as the current bit of the embedded message. The values of *e* and *f* are variable and depend on the pixels of the image used. The smaller the step t, the less likely it is that k will be equal to e or f. If such a situation occurs that k=e or k=f, none of the values of the set $R_{1}$ or $R_{3}$ is suitable for embedding the next bit of the message. In a process of decoding, it will be impossible to determine the presence of such a case, which will create a decoding error due to the loss of a message bit. In the software implementation, we took the step t=0.01, which will reduce the probability of this error and allow us to obtaina sufficient sample of $S_{1}$ and $S_{3}$. But no matter how small the step t is, the number of values of the sets $R_{1}$ and $R_{3}$ will always be limited, since they are rounded values calculated by Equation (1), and even with the maximum possible number of points of the Bezier curve constructed, we will get several equal values. Therefore, it is worth thinking about the quality of the source containers here. If we consider a bitmap image in which there are blocks with only the same brightness, we will inevitably encounter the loss of the embedded bit of the message. Thus, for unambiguous decoding, it is necessary to provide for cases of obviously unsuitable blocks of pixels that will be skipped. The LIBC5 algorithm was launched for a set of 800 grayscale bitmaps with a size of 450 by 450 points, and the embedded messages representing a pseudo-random sequence were successfully decoded. The Bezier curve was plotted in increments of t=0.01.

By replacing the values obtained by Lagrange interpolation in the pixel matrix, we do not violate the overall pixel statistics of the image. At the same time, using the Bezier curve, we use a smooth transition of pixel values, minimizing possible distortions.

Thus, you can fill in up to 50% of the pixels of the image with embedded information. But since the percentage of embedding remained at 21% in the previously studied methods [27,28,29], we embedded approximately the same amount of information for comparative analysis.

## 4. LIBC5 Algorithm Analysis

Next, we conducted a stegoanalysis of the developed LIBC5 method. For the experiment, a set of 800 images with a size of 450 × 450 pixels was used, filled with the proposed method by 21%. The message to be embedded was obtained using a pseudo-random number generator, and then it was encrypted with Vernam cipher. 

The analysis of the obtained stegocontainers was carried out by the RS (regular–singular) method [30]. RS analysis uses a sensitive method of double statistics derived from spatial correlations in images. In the RS method, there are three main factors that affect the accuracy of the estimated message length: the initial deviation, the noise level of the container image and the placement of the message bits in the image. This method shows a fairly accurate result even on noisy images. The experiments carried out by the RS method showed the stego stability of the LIBC5 algorithm. Table 2 shows the result of RS analysis on a set of empty containers, and Table 3shows the result of RS on filled containers using the LIBC5 method, where *L* shows the percentage of information detected. The obtained results of calculating the *type I error* shown that its probability is about 0%. The percentage of detection of the embedded information by the RS method showed the absolute stability of the LIBC5 method developed by us in relation to this type of stagoanalysis.

We will compare the results of the developed method with the INMI (improved neighbor mean interpolation) steganographic method, which we investigated in the article [28]. In [31], a modification of the INMI method was previously obtained based on the use of a Lagrange interpolation polynomial of the second degree to obtain a container image. The Lagrange interpolation formula has the following form:

(11) $$L(x)=\sum_{i=0}^{n}y_{i}p_{i}(x)$$

where $p_{i}(x)$ is the polynomial of degree n, taking a value equal to one, in the node of $x_{i}$, equal to zero—in other nodes $x_{k}$,$k\neq i,k,i=\bar{0,n}$.

In the modified algorithm of the INMI method, the image obtained by adding additional rows and columns of pixels to the original image was considered in fragments of five pixels numbered from 0 to 4. Known pixels (0, 2, 4) are considered interpolation nodes. Therefore, unknown pixel values (1 and 3) were found using the Lagrange interpolation polynomial of degree 2. The image obtained by adding additional rows and columns of pixels to the original image was considered in fragments of five pixels, numbered from 0 to 4. 

The pixel values of the container image are obtained using the following formula:

(12) $$C_{k}=C_{0}\frac{\left(x_{k}-2)\cdot (x_{k}-4\right)}{8}+C_{2}\frac{x_{k}(x_{k}-4)}{-4}+C_{4}\frac{x_{k}(x_{k}-2)}{8},$$

where k=1, 3 is the pixel number in a fragment of five pixels.

Based on the INMI algorithm considered, in [28] we determined that the maximum container capacity is 21% and depends on the image. We conducted research on a set of 800 images of size 450 by 450, that is, using the same set of stegocontainers as in this study of the LIBC5 method. Here are the resulting tables of the stegoanalysis of the INMI method:

Comparing the results obtained using the LIBC5 method in Table 2 and Table 3, and the results of the INMI method in Table 4 and Table 5, we can conclude that LIBC5 is more resistant to steganalysis and can be successfully used on image files forthe tasks of embedding DWM (digital watermarks).

## 5. Conclusions

In this paper, the existing current approaches to embedding watermarks in images were considered and the LIBC5 watermark embedding method was developed and investigated. Special cases and limitations of the method were considered. We have reviewed in detail the developed LIBC5 algorithm, also considering the special case and limitations of the method. The final comparative results of the RS analysis of the INMI and LIBC5 methods were presentedandshow an improvement in the stability indicators of the LIBC5 method compared to INMI. Both methods use the Lagrange interpolation algorithm and are suitable for use in the tasks of embedding DWMs in raster images. The LIBC5 method is not only resistant to the RS method but is also comparable in durability and capacity to the stegosystem of the permutation method for bitmaps discussed in [32], which is a reliable scheme for embedding copyright messages in images.

The further development of the LIBC5 method is aimed to increase the information protection level. For this, it is possible to select a point of the Bezier curve when embedding and extracting, in which the bit that coincides with the date bit is not the lowest but, for example, the third and most significant, and will not affect the quality of the method in any way.

## Figures and Tables

**Table 1:** Turn-based values for a group of five pixels.

t	$R_{k}$	P
0	46	$P_{0}$
0.05	48	$P_{1}$
0.1	49	$P_{1}$
0.15	51	$P_{1}$
0.2	52	$P_{1}$
0.25	54	$P_{1}$
0.3	55	$P_{1}$
0.35	56	$P_{1}$
0.4	57	$P_{1}$
0.45	57	$P_{1}$
0.5	58	$P_{2}$
0.55	58	$P_{3}$
0.6	58	$P_{3}$
0.65	58	$P_{3}$
0.7	58	$P_{3}$
0.75	57	$P_{3}$
0.8	56	$P_{3}$
0.85	55	$P_{3}$
0.9	54	$P_{3}$
0.95	53	$P_{3}$
1	51	$P_{4}$

**Table 2:** RS analysis on a set of empty containers (800 images).

L		0%	1–4%	5% and More
Fileshare	450 × 450	61.25	38	0.5

**Table 3:** RS analysis on a set of filled containers (800 images).

L		0%	1–4%	5% and More
Fileshare	450 × 450	84.75	15.25	0

**Table 4:** RS analysis on a set of empty 450 × 450 containers (800 images).

L		0%	1–4%	5% and More
Fileshare	450 × 450	61	39	-

**Table 5:** RS analysis on a set of 450 × 450 containers filled with 12% interpolation method.

L		0%	1–4%	5% and More
Fileshare	450 × 450	51.5	48	0.5

## Data Availability

Not applicable.
